# Multispecies allometric models for estimating aboveground biomass in plantation and natural dry Afromontane forests in northcentral Ethiopia

**DOI:** 10.1371/journal.pone.0322025

**Published:** 2025-05-07

**Authors:** Getabalew Teshome Reta, Motuma Tolera, Mulugeta Mokria

**Affiliations:** 1 Wondo Genet College of Forestry and Natural Resources, Hawassa University, Shashemene, Ethiopia; 2 Ethiopian Forest Development (EFD), Addis Ababa, Ethiopia; 3 Centre for International Forestry Research and World Agroforestry Centre (CIFOR-ICRAF), Addis Ababa, Ethiopia; Tennessee State University, UNITED STATES OF AMERICA

## Abstract

Dry Afromontane forests in Ethiopia are crucial for carbon sequestration; however, the absence of robust biomass and carbon stock estimation models hinders accurate assessment. This study addresses this limitation by developing and validating site-specific, multispecies biomass estimation models for Wof-Washa plantation and natural forests. Biometric data were collected from 127 harvested trees representing seven dominant species from both plantation and natural forests. Aboveground biomass (AGB) was regressed against diameter at breast height (DBH) as the sole predictor, with stepwise inclusion of total height (H), crown area (CA), and wood density (ρ). Weighted nonlinear least squares regression was performed to fit new models for each forest, their performance was evaluated using the root mean square error (rRMSE), pseudo-R^2^, and relative mean prediction error (rMPE %). The best-selected model using DBH and H explained 90% and 95% of the variation in the AGB of plantation and natural forests, respectively. This model produced the lowest bias (rMPE = 5.9% for plantation and 2.5% for natural forests) compared to pan-tropical models. Our findings demonstrated that our optimal model provides accurate AGB predictions at plot and landscape levels. This confirms that the models can provide sufficiently reliable estimations of carbon stocks, indicating the potential for national carbon accounting and thereby enhancing decision-making in the study forests. Therefore, the findings of this research contribute directly to enhancing the accuracy of carbon dynamic monitoring and supporting sustainable forest management, a crucial component in global efforts to combat climate change.

## Introduction

Tropical forests, which constitute approximately 45% of global forests, are highly diverse and productive ecosystems on earth [1,2]. They provide multiple ecosystem services and functions, including regulating the Earth’s climate by capturing atmospheric carbon dioxide (CO_2_) [3,4]. However, these forests have been experiencing deforestation and forest degradation, which contributes to approximately 15% of the annual global CO_2_ emissions [5] and has adverse impacts on forest ecosystems [6]. As climate change persists rapidly, tropical forest degradation is becoming more complicated, raising concerns among local and global communities [7].

To combat the degradation of tropical forests, it is crucial to implement context-specific measures such as preserving existing forests and establishing smaller to larger plantation areas [8]. Currently, plantation forests and woodlots are becoming sources of wood products and contributing to economic development. They also play a key role in reducing anthropogenic pressures on natural forests and regulating atmospheric CO_2_ through carbon sequestration [6]. There is currently a growing interest in the expansion of plantations through afforestation and reforestation projects [9,10]. This is linked to the global “Reducing Emissions from Deforestation and Forest Degradation” (REDD+) initiative to mitigate climate change [11].

Despite efforts to increase tree cover across the landscape, there is a lack of accurate and reliable information showing the management and utilization of forests [12] and their potential to accumulate carbon and mitigate the anthropogenically driven impacts of climate change [13]. On the other hand, accurate estimation of the aboveground biomass (AGB) and C-stock of trees is crucial for understanding their contributions to national and global carbon (C) budgets and supporting sustainable forest management [13,14]. Reliable forest biomass and C-stock information are important to develop successful forest strategies and policies, as well as for claiming performance-based carbon credits under REDD+ [15]. Thus, there is a national and global interest in developing a robust biomass estimation model to provide accurate and site-specific biomass and carbon stock information [16].

Currently, the generalized biomass estimation model has been widely used in response to the lack of site- and species-specific biomass estimation models [17,18]. This has been acknowledged in the context of the Kyoto Protocol [6] and considerably contributed to biomass data availability, particularly in the tropics [19,20]. Although the accuracy of AGB estimation has not reached the required level, especially in sub-Saharan Africa (SSA) [21], the primary cause of the persistent inaccuracy is the scarcity of site-specific models for biomass estimation that can adequately represent the diverse species composition and tree size variations within the study population [13]. Therefore, applying generalized models outside of areas that reflect their respective vegetation contexts may cause large uncertainties in biomass estimation [22,23]. Therefore, additional tests are necessary to ascertain whether these methods can be applied to other forest biomes [24]. On the other hand, developing site- and species-specific allometric models have been recommended for better estimation of forest biomass and carbon fluxes [25].

Despite their importance, very few allometric biomass estimation models have been developed for sub–Saharan Africa, including Ethiopia. Site- and species-specific allometric models developed in Ethiopia are negligible compared with the species and agroecological diversity of the country [25,26]. Thus, the country relies on generalized and pantropic models [17,27] to report the emission factors of all vegetation types [28]. Although pantropical models contribute to biomass data availability, there is also associated uncertainty in the estimation due to the lack of tree species from Ethiopia when the model is developed. Thus, it is urgent and timely to develop site-specific allometric equations for mixed-species forest stands and to investigate carbon dynamics in the natural dry Afromontane and plantation forests. Therefore, this study aims to derive various mixed-species allometric equations representing dominant tree species growing in the natural dry Afromontane and plantation forests of northcentral Ethiopia.

## Materials and methods

### Site description

**Fig 1:**
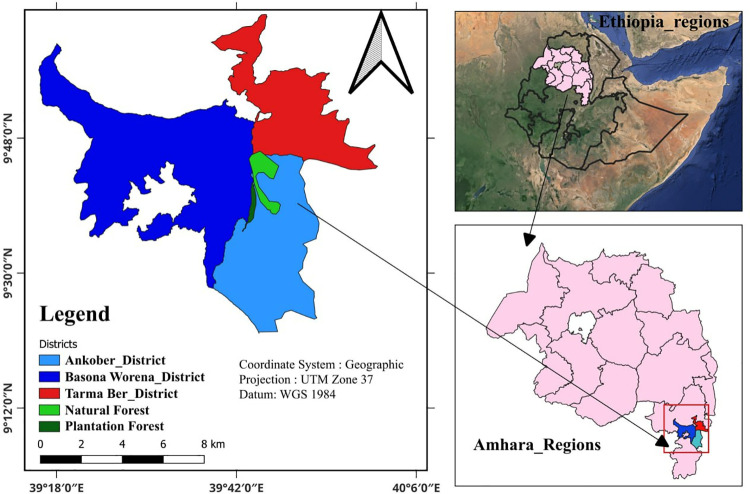
Map of the study site. The map was created using freely obtained shapefiles from the Ethiopian Mapping Agency.

This study was conducted in the Wof-Washa dry Afromontane natural and plantation forests of northcentral Ethiopia. It is located between 9° 44′ to 9°46′ N latitude and 39° 44′ to 39° 47′ E longitude, with altitudes ranging between 1700 and 3700 m above sea level [29] (Fig 1). The study area receives an average of 1,400 mm of rainfall per year with bimodal rainfall patterns, where the long rainy season occurs between July and September and the short rainy season occurs from March to May [30]. The general classification of the soil across the landscape is black clay soil and compact clay soil, as well as reddish-brown heavy loam soil [31].

Originally, the studied natural forest (i.e., “Wof-Washa forest”) is characterized by dry Afromontane mixed broad-leaved and conifer forests [32]. The upper part of the forest is dominated by *Erica arborea* and *Hypericum revolutum*. The southern parts of the forest are dominated by *Juniperus procera*, *Podocarpus falcatus*, *Hagenia abyssinica*, *Allophylus abyssinica* and *Olea europaea* are the dominant species [29]. However, these forests have been under exploitation because of extensive deforestation, fires, and overgrazing [31]. Currently, the forest covers an area of 7550 ha [33] of which about 740 ha is man-made forest and the remaining is natural forest. Plantation establishment around the periphery of the Wof-Washa natural forest began in the early 1980s intending to reduce anthropogenic pressure on the natural forest and meet the increasing demand for wood among the growing population. Most plantations are composed of monocultures of exotic species, particularly from genera of *Eucalyptus, Cupressus, Pinus*, and *Acacia.* The local environment seems to be well suited for these species, which have shown positive outcomes and are considered socially acceptable. However, appropriate forest management practices have not been properly implemented to enhance the productivity of this plantation [34].

### Data collection and sampling techniques

#### Vegetation data collection.

We conducted a reconnaissance survey across the plantation and natural forests of the Wof-Washa based on information from the North Shewa Agricultural Office to select a landscape encompassing both forests. Hence, plantation forest comprising *Eucalyptus globulus*, *Cupressus lusitanica*, *Pinus patula* stands and the adjacent natural forest were selected for this study. We used a systematic transect sampling technique to collect vegetation data [35]. The first sample plot was randomly located, followed by systematic placement of the other plots within an equal interval in each transect within each stand [35]. The number of plots per transect and transect lines per landscape was established depending on the size and form of the landscape encountered. In total, we established 119 square plots (20 m × 20 m in size), that is, 79 plots were established in natural forest, whereas 40 plots were in plantation forests. The plantation sample plots included E*. globulus* (14 plots), C. *lusitanica (*14 plots*) and P. patula* (12 plots) stands. All sample plots were laid at least 30 m away from the stand edge or road to avoid edge effects [36].

In each plot, the species name, diameter at breast height (DBH), total height (H), widest crown diameter (CD1), and perpendicular crown diameter (CD2) of all trees with a height > 2 m and DBH ≥ 2.5 cm were recorded. Additionally, we recorded stem density and calculated basal area in each plot to ensure the selection of representative sample trees encompassing a wide diameter range (Table 1 and refer the S1 Table for further details). Elderly residents in the study area provided the vernacular names of the recorded woody species. The botanical nomenclature for each species was documented using expert knowledge and published resources. Based on the pilot inventory data, seven dominant tree species (3 species from plantation and 4 species from natural forest) were selected for this study. The selected tree species represented *Eucalyptus globulus, Cupressus lusitanica* and *Pinus patula* from the plantation stands*,* while *Juniperus procera*, *Podocarpus falcatus (Thunb.)*, *Olea europaea*. *subsp. cuspidate*, and *Rahus natalensis (Krauss.)* from natural forest. We categorized the vegetation inventory data into six diameter classes to determine the tree size distribution of the study population. Subsequently, we randomly chose 127 sample trees (69 from plantation and 58 from natural forest) proportional to the size-class distribution for AGB estimation (Table 2).

**Table 1:** Summary of plot inventory data from studied plantation and natural forests.

Forest stands	Age (yr)	N	Stem density (plot^-1^) ± S.E	Mean DBH (cm) ± S.E	Mean H (m) ± S.E	Mean CA (m^2^) ± S.E	Mean BA (m^2^ha^-1^) ± S.E
*E. globulus*	*42*	*336*	24.07 ± 1.17	24.3 ± 0.72	26.5 ± 0.76	9.3 ± 0.53	35.95 ± 0.02
C. *lusitanica*	42	332	23.36 ± 1.23	25.24 ± 0.57	21.48 ± 0.31	3.31 ± 0.18	34.62 ± 0.01
*P. patula*	*42*	*306*	25.25 ± 1.85	32.18 ± 0.61	28.84 ± 0.48	7.1 ± 0.37	57.45 ± 0.02
Natural forest	n/a	1748	22.05 ± 0.56	21.61 ± 0.51	14.71 ± 0.26	n/a	39.83 ± 0.02

SE refers to the standard error of the mean for each variable; N indicates the total observed individual trees per stand; and n/a = the data is not available here.

**Table 2:** Distribution of harvested trees across tree size used in biomass estimation models.

Forests	Species	DBH classes (cm)						
**≤ 10**	**10.1–20**	**20.1–30**	**30.1–40**	**40.1–50**	**≥ 50.1**	**Total**
Plantation forest	*Eucalyptus globulus*	1	3	4	4	7	6	25
	*Cupressus lusitanica*	1	4	7	7	4		23
	*Pinus patula*	1	3	8	5	4		21
**n**		**3**	**10**	**19**	**16**	**15**	**6**	**69**
Natural forest	*Juniperus procera*	2	3	2	3	3	2	15
	*Podocarpus falcatus*	1	2	4	2	2	2	13
	*Olea europaea*	1	4	4	5	1	0	15
	*Rahus natalensis*	2	3	3	5	2	0	15
**n**		**6**	**12**	**13**	**15**	**8**	**4**	**58**

n = number of harvested individual trees per tree size class.

#### Destructive sampling procedures.

Before harvesting the selected sample trees, the biometric properties of each tree were recorded, including DBH (diameter at breast height measured at 1.3 m above the ground), DSH (diameter at stump height at 30 cm above the ground), total height (H), widest crown diameter (CD1), and perpendicular crown diameter (CD2). DBH and DSH were measured with a calliper. The crown diameters (CD1 and CD2) were converted to the crown area (CA) using the method described in Tetemeke [37]. Next, all the sampled trees were harvested at the DSH level using a chainsaw, and their corresponding heights were measured using measuring tape. The stump heights and their respective diameters were also measured for stump volume and dry weight determinations as described by Basuki [15]. For sampling purposes, harvested wood components were dissected into merchantable stems (stems ≥ 10 cm), large branches (branches with a diameter ≥ 2 cm and stem tops with a diameter < 10 cm) and foliage (small branches with a diameter < 2 cm, twigs and leaves). For further local wood processing, the merchantable stems were tracked to the minimum commercial length of 2.1 m intervals up to a minimum top diameter of 10 cm, whereas those < 10 cm in diameter were considered branches. All fresh logs, branches and foliage were subsequently weighed separately in the field via a spring balance (50 and 100 kg capacity).

#### Sub-samples for dry-weight biomass and wood basic density.

To determine the dry weight, three sub-samples were taken from each partitioned tree: stems (i.e., discs from the base, middle and top parts of the stem), branches (small, medium, and large) and foliage (small, medium, and large twigs). The sub-samples were then weighed, labelled, and transported to the Debere Birhan Agricultural Research Centre laboratory for drying. The sub-samples were then oven-dried for 2–3 days at a temperature of 105 °C. The sub-samples were monitored and weighed recurrently every 24 hours until a constant weight was attained [38]. The fresh-to-oven-dry weight ratios were calculated after all the oven-dried samples were weighed. These ratios were used to convert the total fresh weights of sample trees measured in the field into total oven-dry weights [25,39].

To determine the basic wood density, separate wood sub-samples were collected from four points along the tree’s height: the bottom (30 cm above ground level), diameter at breast height (DBH), the midpoint between DBH and the upper stem, and the top of the stem [40]. The basic wood density (g.cm^-3^) was determined using water displacement techniques [41]. To do this, small wood subsamples were taken from each stem and branch disc in three dimensions to determine their green volume using water displacement method. The extracted samples were weighed and labelled, and their green volumes were determined by measuring the displaced water (green volume in cm^3^). The measured fresh weights of the sample discs were converted to their respective green volumes. Afterwards, the samples were oven-dried in the laboratory for 72 hours at 105 °C to determine their dry mass. Thus, the basic wood density (ρ) was calculated from the average dry weight-to-fresh volume ratio [41].

### Biomass estimation model development and evaluation

The response variable was the total oven-dry AGB (AGB in kg), i.e., the sum of merchantable stem, branch and foliage biomass (dry weight) of the harvested sample trees. Before the models were developed, the data were grouped into two categories: Group I, a sample from the plantation forest (n = 69); and Group II, a sample from the natural forest (n = 58). The above-ground biomass estimation models were developed using non-linear regression equations based on stem diameter (DBH in cm), total tree height (H in m), crown area (CA in m^2^), and basic wood density (ρ in g.cm^-3^) [17,25]. Notably, using multiple predictors simultaneously can result in collinearity issues, which may negatively impact the precision of the regression coefficients [42,43]. To address this, a multicollinearity test using Variance Inflation Factors was conducted, with a VIF value exceeding 10 indicating significant collinearity and suggesting that the use of both parameters should be avoided [25]. Ultimately, separate predictive biomass estimation models were developed for each forest. Generally, eight model forms were tested to select the best combination of predictor variables based on either DBH alone or combined with stepwise inclusion of H, ρ and CA while developing predictive biomass estimation model (Table 3).

**Table 3:** Model forms used to predict tree biomass.

Predictors(s)	label	Model eqs
DBH	M1	AGB = β0 Χ (DBH)^β1^
DBH and H	M2	AGB = β0 Χ (DBH)^β1^ Χ (H)^β2^
DBH and ρ	M3	AGB = β0 Χ (DBH)^β1^ Χ (ρ)^β2^
DBH and CA	M4	AGB = β0 Χ (DBH)^β1^ Χ (CA)^β2^
DBH, H and ρ	M5	AGB = β0 Χ (DBH)^β1^Χ (H)^β2^ Χ (ρ)^β3^
DBH, H and CA	M6	AGB = β0 Χ (DBH)^β1^ Χ (H)^β2^ Χ (CA)^β3^
DBH, ρ and CA	M7	AGB = β0 Χ (DBH)^β1^ Χ (ρ)^β2^ Χ (CA)^β3^
DBH, H, ρ and CA	M8	AGB = β0 Χ (DBH)^β1^ Χ (H)^β2^ Χ (ρ)^β3^ Χ (CA)^β4^

We used a weighted nonlinear least-squares regression technique using the ‘nls’ function in R to fit the models. Weighting by 1/(DBH)^2δ^ was performed to account for heteroscedasticity in the residuals [44]. The value of δ represents a weighting factor, which was determined by following the procedures of Picard et al. [45]. Model diagnostics were employed using the metrics of various goodness-of-fit statistics. These included the percent relative standard error (PRSE) (Eq. 1), the Akaike information criterion (AIC) (Eq. 2), the mean absolute prediction error (MAPE) (Eq. 3), and the pseudo-R^2^ (Eq. 4). The best model should have the lowest values for PRSE, AIC, and MAPE while concurrently showing the highest pseudo-R^2^ value [46]. As the number of parameters increases, a model typically exhibits lower RMSE and higher R^2^ values, regardless of their contribution to explaining the response variable’s variation [47]. In this case, the Akaike Information Criterion (AIC) was generally employed to select the final models, as it considered the number of parameters and corrected them accordingly [27]. A model is also considered unreliable when the PRSE exceeds 25% for any of its parameters [42]. Thus, each model was assigned a rank based on individual goodness-of-fit statistics; these ranks were summed, and the sums were ranked to determine an overall performance ranking for the models [48]. These statistical parameters were calculated as follows:


$$\begin{array}{c} PRSE=\left(SE/|\beta |\right)x100 \end{array}$$ (1)

Where PRSE is the percent relative standard error; SE is the standard error of the parameter estimates and|β| refers to the absolute value of the parameter.


$$A|C=n|n\left(\frac{\sum_{i=0}^{n}y-{\overset{´}{y}}_{i}^{2}}{n}\right)+2p$$ (2)

$$\begin{array}{c} MAPE(\%)=\frac{100}{n}\sum_{i=1}^{n}\left(\frac{y_{i}-{\overset{´}{y}}_{i}}{y_{i}}\right) \end{array}$$ (3)

$$\begin{array}{c} Pseudo{-\text{R}}^{2}=1-\frac{\sum_{i=1}^{n}\;{\left(y_{i}-{\overset{´}{y}}_{i}\right)}^{2}}{\sum_{i=1\;}^{n}{\left(y_{i}-{\overset{´}{y}}_{i}\right)}^{2}} \end{array}$$ (4)

Where y_i_ is the observed individual AGB; ỹ is the mean observed AGB; ýi is the predicted individual AGB; n is the number of observations; and p is the number of model parameters.

We conducted a cross-validation process to validate and select the best-fitting model for each group (plantation and natural forests) [49]. In principle, the allometric models should be validated using an independent dataset; however, such data was not available for the plantation and natural forests in our study area. Thus, we used a validation set approach [50], dividing the total sample trees (for plantation: n = 69; and natural forest: n = 58) into two subsets. For model calibration, we utilized 49 sample trees from the plantation and 42 trees from natural forests, whilst the remaining 20 plantation trees and 16 natural forest trees were used for model validation (testing set). The partitioning of the data set was done based on the size class distribution of harvested sample trees.

The goodness-of-fit statistics and the coefficients from the ‘training’ models were compared with those obtained using the full dataset. The model evaluation was carried out using the root mean square error (RMSE) (Eq. 5), mean absolute error (MAE) (Eq. 6) and mean prediction error (MPE) (Eq. 7) [51]. These model performance indicators and their respective relative values were computed as:


$$\begin{array}{c} RMSE(kg)=\sqrt{\frac{1}{n}}\sum_{i=1}^{n}{\left(y_{i}-{\overset{´}{y}}_{i}\right)}^{2};rRMSE(\%)=\frac{RMSE}{\tilde{y}}\times 100 \end{array}$$ (5)

$$MAE(kg)=\left(\sum_{i=1}^{n}\frac{\left|y_{i}-{\overset{´}{y}}_{i}\right|}{y_{i}}\right);rMAE(\%)=\frac{MAE}{\tilde{y}}x100$$ (6)

$$MPE(kg)=\sum_{i=1}^{n}\left(\frac{\left(y_{i}-{\overset{´}{y}}_{i}\right)}{n}\right);rMPE(\%)=\frac{MPE}{\tilde{y}}x100$$ (7)

Where y_i_ is the observed individual AGB; ỹ is the mean observed AGB; ýi is the predicted individual AGB; n is the number of observations.

The relative mean prediction error (rMPE%) is a key indicator of whether the model satisfies the expected accuracy requirements. This is a measure of the systematic deviation of the model estimations from the observed data. It is an important and widely used statistical parameter for model evaluation in addition to the 95% confidence interval of the predictions [52–54]. A relative mean prediction error (rMPE) <± 10% at the 95% confidence level was considered acceptable, according to [55]. Accordingly, the MPE was calculated for all the validated models, and then a simple t-test was used to test whether the MPE was significantly different from zero. After that, the full dataset (for Group I: n = 69; and Group II: n = 58) was used to develop the final models. Finally, we compared the performance of our best AGB models of plantation and natural forests with generalized pantropical models developed by Brown [18] and Chave et al. [17].

### Assessing bias in aboveground biomass estimation

To assess the bias introduced in AGB estimation associated with species aggregation in multispecies models, we compared the plot AGB estimate derived from the multispecies model with the estimate obtained from the species-specific allometric model, following the methodology outlined by Van-Breugel [56]. To assess the relative errors in predictions from mixed-species models, we used AGB estimates derived from species-specific models developed by Reta et al. (unpublished manuscript) for the species *E. globulus*, *C. lusitanica*, and *P. patula* in the Wof-Washa plantation forest. This analysis also included the mixed-species models developed in this study for plantation forest, as well as pan-tropical models developed by Brown [18] and Chave et al. [17].

We employed several calculations to evaluate the bias in AGB estimation using multispecies models. These included determining the relative error for individual plot AGB estimates (RE_plot, i_) (Eq. 8), the relative error plot-level AGB estimates (RE_plot-level_) (Eq. 9), and the mean relative error across all plot estimates (RE_across-plot_) (Eq. 10). We subsequently established confidence intervals (CIs) for plot-level AGB estimations produced from species-specific models. This process involved the following key steps: Initially, we computed the relative standard error (RSE) (Eq. 11) for each species-specific model, followed by calculating the standard error (SE_i_) (Eq. 12) for each species AGB estimation. We then determined SE_plot_ (Eq. 13) for sets of uncorrelated variables with differing variances. The following is how these errors were calculated:


$${RE}_{plot,i}=100x\frac{\left({AGB}_{ss,i}-{AGB}_{ms,i}\right)}{{AGB}_{ss,i}}$$ (8)

$${RE}_{plot-level}=\frac{100}{n}\sum_{i=}^{n}\left(|{AGB}_{ms,i}-{AGB}_{ss,i}|/{AGB}_{ssi}\right)$$ (9)

$${RE}_{across-plot}=100x|\left(\frac{1}{n}\sum_{i=1}^{n}{AGB}_{ms,i}\right)-\left(\frac{1}{n}\sum_{i=1}^{n}{AGB}_{ss,i}\right)|/\frac{1}{n}\sum i=1{AGB}_{ss,i}$$ (10)

Where RE_plot, i_ = relative error of each plot AGB estimate; AGB_ms, i_ = AGB estimates of multispecies models; AGB_ss,_ i = AGB estimates of species-specific models. RE_plot-level_ = relative error of plot_-_level AGB estimates; RE_across-plot_ = mean relative error across-plot average of AGB estimates and n is the number of plots.


$$\begin{array}{c} \text{RSE}=\sqrt{\left(\exp\left(\text{MSE}/(\text{n}-\text{k)}\right)-1\right)} \end{array}$$ (11)

Where RSE is the relative standard error; MSE is the regression mean squared error; n is the number of individuals, and k is the number of model parameters.


$$\begin{array}{c} {\text{SE}}_{\text{i}}={\text{RSE}}_{\text{i}}\times {\text{AGB}}_{\text{i}} \end{array}$$ (12)

Where SE_i_ is the standard error for the AGB estimation of a specific species


$$\begin{array}{c} {\text{SE}}_{\text{plot}}=\sqrt{\left(\sum \left({\text{SE}}_{1}^{²}\ldots {\text{SE}}_{\text{n}}^{²}\right)\right)} \end{array}$$ (13)

Where SE_plot_ is the standard error of the plots for sets of uncorrelated variables with various variances.

Finally, we established 95% confidence intervals for the AGB estimates from the species-specific models using the following formula: estimated plot AGB ± 1.96 × SE_plot_, using data from 40 plantation forest plots (refer the S1 File for further details). AGB estimates from the multispecies models were deemed significantly different if they fell outside this 95% confidence interval. All data analyses were conducted using R version 4.3.2 (R Core Team, 2023).

## Results

### Sampled species and their biometric relationships

The harvested dominant tree species, their dendrometry information (DBH, H, ρ and CA) and the range of oven-dry biomass per species are presented in Table 4. The correlations between AGB and DBH as well as between AGB and H were significantly positive (*P < 0.001*) for both plantation and natural forests (S1a,b,e,f Fig in supporting information). The relationships between AGB and ρ were weak compared to other parameters (S1d,h Fig).

**Table 4:** Tree species sampled and their mean of dendrometric properties for biomass modelling.

Species name	n	Dendrometric parameters				Oven dry biomass of measured tree components (Kg, tree)			
		DBH (cm)	H (m)	ρ (g.cm^-3^)	CA (m^2^)	Stem	Branch	Foliage	AGB (kg)
		Mean ± S.E	Mean ± S.E	Mean ± S.E	Mean ± S.E	Mean ± S.E	Mean ± S.E	Mean ± S.E	Mean ± S.E
*Eucalyptus globulus*	25	37.73 ± 3.11	28.15 ± 1.98	0.54 ± 0.01	16.35 ± 2.16	866.29 ± 151.60	69.59 ± 8.82	17.19 ± 2.35	953.08 ± 158.28
*Cupressus lusitanica*	23	29.18 ± 2.16	21.61 ± 1.58	0.45 ± 0.02	6.14 ± 0.85	171.08 ± 36.18	37.91 ± 5.93	15.06 ± 2.21	224.05 ± 42.75
*Pinus patula*	21	30.3 ± 2.48	23.93 ± 1.93	0.44 ± 0.02	7.24 ± 1.66	333.77 ± 73.10	19.75 ± 2.99	12.48 ± 1.96	366.01 ± 77.75
*Juniperus procera*	15	30.04 ± 4.06	20.34 ± 2.45	0.55 ± 0.01	53.92 ± 9.36	355 ± 107.28	71.33 ± 14.33	21.65 ± 3.96	447.98 ± 124.44
*Podocarpus falcatus*	13	32.05 ± 4.57	22.22 ± 2.75	0.54 ± 0.02	50.84 ± 11.46	431.35 ± 148.67	82.36 ± 18.82	18.64 ± 3.57	532.35 ± 169.61
*Olea europaea*	15	26.11 ± 2.60	15.75 ± 1.20	0.51 ± 0.01	22.84 ± 4.56	148.78 ± 21.93	124.72 ± 27.35	55.29 ± 8.39	328.78 ± 55.39
*Rahus natalensis*	15	25.62 ± 3.03	18.35 ± 1.32	0.53 ± 0.01	16.43 ± 50	196.81 ± 54.18	34.92 ± 5.39	10.28 ± 1.71	242 ± 60.44

SE refers to the standard error of the mean for each variable; n indicates number of individual trees per species.

### Developed multispecies allometric models

The parameter estimates of the AGB models with their fit statistics are presented in Table 5. The model (M1), which used DBH as the predictor, explained 88.7% and 90.8% of the variation in AGB for plantation and natural forests, respectively. However, this model exhibited a greater relative root mean square error (rRMSE), mean absolute percentage error (MAPE), and Akaike Information Criterion (AIC) than the other valid models did. When tree height (H) was included in the DBH-alone model, the parameters of model M2 were highly significant (p < 0.001) with a percent relative standard error (PRSE) of the coefficients < 25% for both plantation and natural forests. The model explained 90% and 95% of the variation in AGB for plantation and natural forests, respectively. This model (M2) exhibited less error, with a mean absolute percentage error (MAPE) of 28.5% and 24.1% for plantation and natural forests, respectively. Despite the inclusion of crown area (CA) with DBH and H in the model enhanced some fit statistics, this model produced a higher PRSE value (PRSE exceeds 25%) for both plantation and natural forests. However, the incorporation of wood density (ρ) into the models led to non-significant model parameters and inflated PRSE for both forests (Table 5). Thus, the inclusion of wood density (ρ) did not improve the predictive capacity of the models for both plantation and natural forests. Therefore, model-fitting statistics analysis showed that the M2 is the best model for both plantation and natural forests.

**Table 5:** Multispecies models developed in this study using different combinations of tree variables.

Models	Parameters					Fit statistics				PRSE				
	β0	β1	β2	β3	β4	R^2^	RMSE (%)	MAPE (%)	AIC	β0	β1	β2	β3	β4
**Group I. Multispecies models using the full dataset from plantation forest (n = 69)**														
M1	0.032^***^	2.671^***^				0.89	40.0	34.8	845.3	35.2	3.8			
**M2**	**0.015** ^***^	**1.216** ^***^	**1.806** ^***^			**0.90**	**37.2**	**28.5**	**821.3**	**23.7**	**21.1**	**18.5**		
M3	0.111^ns^	2.468^***^	0.739^*^			0.87	42.5	33.1	841.9	61.6	5.2	42.9		
M4	0.122^ns^	2.137^***^	0.255^**^			0.91	35.7	33.4	838.3	55.6	9.7	34.6		
M5	0.33^ns^	1.150^***^	1.741^***^	0.444^ns^		0.89	38.7	27.5	820.2	54.4	22.9	19.3	58.0	
M6	0.05^*^	0.688^*^	1.854^***^	0.240^**^		0.92	32.3	26.2	810.6	46.8	41.0	17.0	29.1	
M7	0.30^ns^	2.090^***^	0.518^ns^	0.212^*^		0.90	37.7	31.9	837.4	65.5	9.6	60.4	42.0	
M8	0.072^ns^	0.681^*^	1.822^***^	0.266^ns^	0.221^**^	0.92	33.5	25.9	811.3	56.0	41.8	17.4	32.2	32.2
**Group II. Multispecies models using the full dataset from natural forest (n = 58)**														
M1	0.246^***^	2.113^***^				0.91	33.0	26.7	644.8	22.8	3.2			
**M2**	**0.151** ^***^	**1.778** ^***^	**0.540** ^***^			**0.95**	**24.2**	**24.1**	**640.9**	**21.6**	**8.4**	**22.3**		
M3	0.698^*^	1.991^***^	1.035^*^			0.93	27.8	24.7	642.1	46.8	3.9	42.2		
M4	0.336^**^	1.956^***^	0.067^ns^			0.91	33.5	25.9	645.1	33.2	7.4	84.0		
M5	0.345^ns^	1.776^***^	0.403^ns^	0.664^***^		0.95	24.3	23.4	641.3	62.7	8.5	60.0	72.2	
M6	0.203^**^	1.646^***^	0.522^*^	0.124^*^		0.95	24.7	23.8	641.3	37.2	11.4	41.2	45.1	
M7	0.919^ns^	1.844^***^	1.018^*^	0.066^ns^		0.93	28.1	24.1	642.3	51.5	7.8	42.5	84.5	
M8	0.463^ns^	1.640^***^	0.389^ns^	0.666^***^	0.064^ns^	0.95	24.5	23.1	641.6	66.6	11.5	61.3	71.1	86.0

M refers to the model developed in this study, while the numbers indicate the numbering of the models, as displayed in the Materials and Methods section. α and b are the estimated model scaling coefficients and exponents, respectively. The statistical analyses are significant at the 95% confidence interval.

*** p < 0.001;

** <0.01;

* p < 0.05; and nonsignificant, ns p > 0.05; the viable models with the best model fitting indicators are highlighted in **bold**.

#### Cross-validated AGB models.

The cross-validation results of the viable models for plantation and natural forests are provided in Tables 6 and 7. The model’s parameter values in our viable and cross-validation models remained consistent across subsets of the “test” dataset (Tables 5–7). There were only slight variations in the parameter estimates for coefficients “α” and “b” for the viable models when the complete dataset was used and when the training dataset was used (Tables 5 and 6). The viable model (M1) using DBH alone on the testing dataset generated moderate prediction accuracy, with rMPEs of 8.65% and 7.06% for plantation (in Group I) and natural forest (Group II), respectively (Table 7). Compared with the DBH-alone model (M1), including H improved the model performance, decreasing the rMPE from 8.65% to 1.27% for plantation forest. The viable models further demonstrated that the standard errors of the coefficients (α) and (b) of our best models were not highly inflated, with an acceptable value of PRSE. In addition, the standard error of the estimate is nearly equal to the RMSE from the cross-validation test statistics (Tables 5–7); thus, our best models are reliable for capturing the variations in AGB. Therefore, based on the model performance test results, M2 was ranked as the best model using DBH and H for both plantations and natural forests.

**Table 6:** Variable coefficients and validation metrics of cross-validated models with training set.

Models	Parameters					Validation Indicators						
	β0	β1	β2	β3	β4	R^2^	RMSE (kg)	RMSE (%)	MAE (kg)	rMAE (%)	MAPE (%)	AIC
**Group I. Cross-validated models using the training set of plantation trees (n = 49)**												
M1	0.036^*^	2.634^***^				0.86	249.27	47.2	158.5	30.0	38.8	610.8
**M2**	**0.010** ^**^	**0.760** ^**^	**2.439** ^**^ ^*^			**0.90**	**201.50**	**38.1**	**133.4**	**25.3**	**27.6**	**570.9**
M3	0.099^ns^	2.461^***^	0.573^ns^			0.83	268.11	50.8	165.1	31.3	37.4	610.8
M4	0.125^ns^	2.110^**^^*^	0.277^*^			0.90	211.90	40.1	133.1	25.2	34.0	605.7
M5	0.016^ns^	0.666^**^	2.449^***^	0.286^ns^		0.89	212.54	40.2	132.3	25.1	27.1	571.7
M6	0.022^*^	0.376^ns^	2.475^***^	0.193^**^		0.93	167.62	31.7	110.0	20.8	25.5	564.1
M7	0.141^ns^	2.109^***^	0.112^ns^	0.262^*^		0.89	214.90	40.7	133.4	25.3	33.8	607.6
M8	0.022^ns^	0.371^ns^	2.478^***^	0.028^ns^	0.190^*^	0.93	168.74	31.9	110.2	20.9	25.5	566.1
**Group II. Cross-validated models using the training set of natural forest trees (n = 42)**												
M1	0.079^*^	2.433^***^				0.97	72.02	19.3	55.9	15.0	27.5	459.8
**M2**	**0.095** ^**^	**1.742** ^***^	**0.727** ^*^			**0.97**	**69.89**	**18.8**	**52.9**	**14.2**	**23.6**	**456.2**
M3	0.056^ns^	2.492^***^	0.220^ns^			0.97	70.96	19.0	55.1	14.8	27.8	461.7
M4	0.088^*^	2.375^***^	0.025^ns^			0.97	72.44	19.4	55.8	15.0	27.2	461.7
M5	0.019^ns^	1.643^***^	1.136^**^	1.141^*^		0.98	61.26	16.4	47.8	12.8	24.0	453.4
M6	0.109^ns^	1.673^***^	0.728^*^	0.030^ns^		0.97	70.26	18.9	53.5	14.4	23.5	457.9
M7	0.065^ns^	2.433^***^	0.189^ns^	0.022^ns^		0.97	71.52	19.2	55.2	14.8	27.5	463.5
M8	0.021^ns^	1.614^***^	1.129^**^	1.121^***^	0.013^ns^	0.98	61.72	16.6	47.9	12.8	23.9	455.3

The statistical analyses are significant at the 95% confidence interval.

*** p < 0.001;

** <0.01;

* p < 0.05; and nonsignificant, ns p > 0.05; the best performing model indicators are highlighted in bold.

**Table 7:** Predictive accuracy of the viable multispecies models on the test datasets.

Models	Observed AGB (Kg)	Predicted AGB (Kg)	MSE (kg)	rRMSE (%)	MAE (kg)	rMAE (%)	MPE (kg)	rMPE (%)
Group I. Models of plantation on the testing set (n = 20)								
M1	539.23	492.60	286.18	53.07	107.26	19.89	46.62	8.65^ns^
**M2**	**539.23**	**532.39**	**182.44**	**33.83**	**127.41**	**23.63**	**6.84**	**1.27** ^**ns**^
M3	539.23	497.92	157.23	29.16	100.27	18.60	41.31	7.66^ns^
M4	539.23	496.47	149.44	27.71	104.03	19.29	42.75	7.93^ns^
M5	539.23	534.36	175.34	32.52	119.44	22.15	4.87	0.90^ns^
M6	539.23	532.78	162.00	30.04	111.14	20.61	6.45	1.20^ns^
M7	539.23	498.49	144.65	26.82	101.76	18.87	40.74	7.55^ns^
M8	539.23	533.13	161.48	29.95	110.59	20.51	6.10	1.13^ns^
**Group II. Models of natural forest on testing set (n = 16)**								
M1	409.54	380.64	114.31	27.91	74.47	18.18	28.90	7.06^ns^
**M2**	**409.54**	**380.59**	**103.49**	**25.27**	**66.21**	**16.17**	**28.94**	**7.07** ^**ns**^
M3	409.54	379.99	118.04	28.82	77.09	18.82	29.55	7.21^ns^
M4	409.54	381.25	114.62	27.99	74.65	18.23	28.29	6.91^ns^
M5	409.54	376.32	113.12	27.62	70.72	17.27	33.22	8.11^ns^
M6	409.54	381.23	103.48	25.27	64.40	15.72	28.31	6.91^ns^
M7	409.54	380.60	117.77	28.76	76.90	18.78	28.94	7.07^ns^
M8	409.54	376.63	112.93	27.57	69.79	17.04	32.90	8.03^ns^

P-value indicating that rMPE was significantly different from zero at the 95% confidence interval when

*** p < 0.001;

** p < 0.01;

* p < 0.05; ns p > 0.05; the best-performing model indicators are highlighted in bold.

### Model comparison

**Fig 2:**
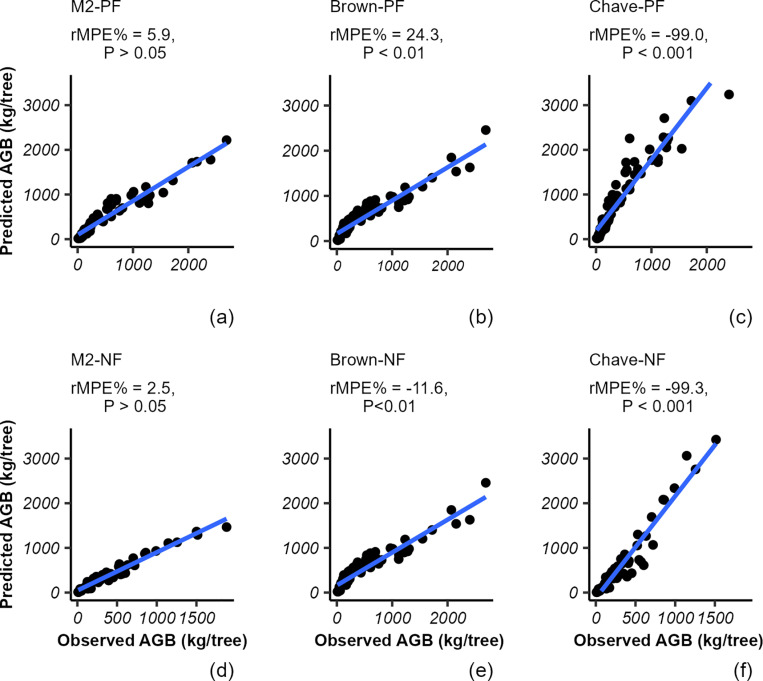
Relationships between predicted and observed aboveground biomass of the harvested trees. The labelled M2-PF (Fig 2a) and M2-NF (Fig 2d) refer to the best-performing AGB models developed in this study for plantation and natural forests, respectively. Pan-tropical models are represented using the first authors’ name: Brown = Brown [18]; Chave = Chave et al. [17]. In Fig 2a–c, the text “PF” indicates the AGB of harvested sample trees (n = 69) from Wef-Washa plantation forest used in testing model performance, whilst “NF” in Fig 2d–f signifies those from the natural forest (n = 58). rMPE (%) is the relative mean prediction error produced in the estimation of AGB. The diagonal lines show a 1:1 relation. Positive and negative rMPE values indicate under- and overestimation of the AGB, respectively. The p-value for rMPE was significantly different from zero at the 95% confidence interval when P < 0.05.

**Fig 3:**
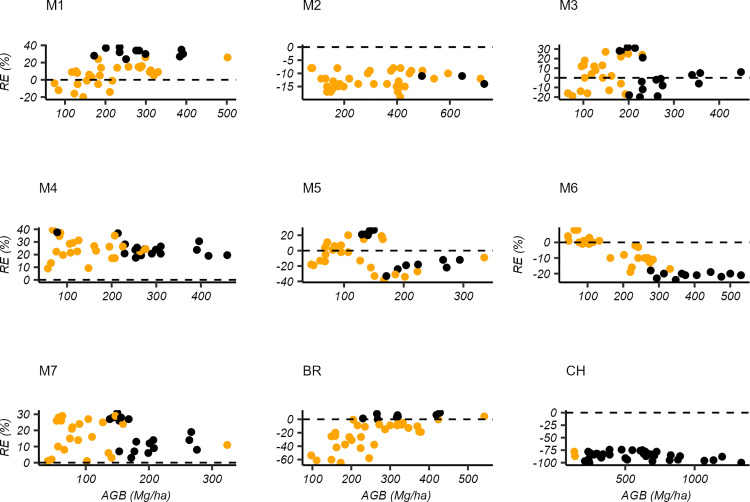
Bias in the AGB estimates of individual plots resulted from the aggregation of species in multispecies models. The x-axis labels M1–7 correspond to multispecies models developed in this study using the dataset of plantation forest (Group I) as shown in Table 5. The remaining letters represent pan-tropical models: BR = Brown [18] and CH = Chave et al. [17]. RE (%) refers to relative errors in %. Orange dots represent AGB estimates from multispecies models that fell within the 95% confidence intervals of species-specific model estimates. Whereas, black dots indicate models whose estimates lie outside this confidence interval.

We plotted the AGB estimated with our best multispecies models of plantation and natural forests and with generalized pantropical models against the observed (measured) tree biomass shown in Fig 2. The observed AGB reflected the trend of the predicted AGB from the best model developed in this study (Fig 2a,d). Compared with the errors from the models of Brown [18] and Chave et al. [17], our model produced the lowest relative mean prediction error (rMPE) of 5.9% and 2.5% for plantation and natural forests, respectively. The application of Brown’s [18] model to each forest dataset resulted in significant errors (rMPE% = -11.6–24.3%, P < 0.05 (Fig 3b,e)). This model overestimated the AGB by 3.9% and 11.6% in plantation and natural forests, respectively. Similarly, the model developed by Chave et al. [17] produced large errors (Fig 2c,f) and markedly overestimated the AGB by 99% and 99.3% for plantation and natural forests, respectively. Therefore, our results demonstrate vegetation- and site-specific allometric models provided more accurate AGB estimates than generalized models did.

### Bias in AGB estimation using multispecies models

**Fig 4:**
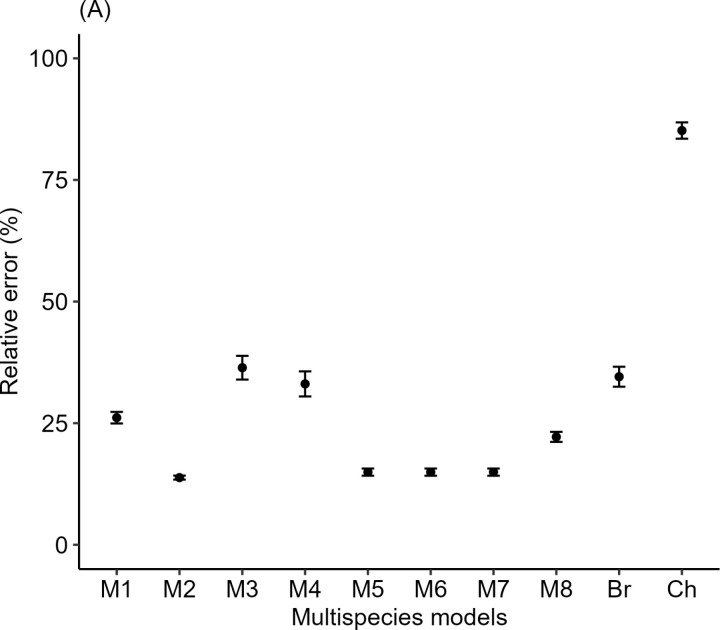
The mean relative error in plot-level AGB estimates resulted from the aggregation of species multispecies models. Mean relative errors of the plot-level AGB estimates are represented by black dots; error bars show the 95% confidence interval of the mean. The x-axis labels M1–8 correspond to multispecies models developed in this study using the dataset of plantation forest. The remaining letters represent pan-tropical models: Br = Brown [18] and CH = Chave et al. [17].

The bias in the plot and landscape AGB estimations associated with species aggregation in multispecies models are illustrated in Figs 3,4 and Table 8. In comparison to the pantropical models of Brown [18] and Chave et al. [17], the multispecies models developed in this study demonstrated that individual plots AGB estimates using plantation models (under Group I) exhibited a minimal bias, with relative errors ranging from 2.3–24.7%. Of the 40 plots, 71.9% fell within the confidence intervals of their respective AGB estimates using species-specific models (Fig 3).

**Table 8:** Bias in across-plot average AGB estimates using data from 40 plots of plantation forest.

Testing dataset	Model forms	Observed AGB (Mg/ha) ± SE	Predicted AGB (Mg/ha) ± SE	RE across plot (%)
Plantation forests (n= 974 trees)	M1	471.16 ± 16.15	386.55 ± 13.99	17.96
	**M2**		**546.86 ± 18.93**	**16.07**
	M3		318.52 ± 9.84	32.4
	M4		381.57 ± 12.22	27.76
	M5		340.38 ± 10.74	24.85
	M6		558.83 ± 20.43	18.61
	M7		343.09 ± 11.94	27.18
	M8		395 ± 15.07	16.2
	Brown 1997		370 ± 13.98	21.47
	Chave et al. (2014)		923.68 ± 35.46	96.04

Observed AGB refer to the average estimate of the landscape AGB (Mg/ha) using species-specific models developed in this study, whereas, predicted AGB is biomass estimates using the given local mixed-species and pantropical models. SE denotes the standard error.

Similarly, the plantation model performed best in estimating plot-level AGB, with a mean relative error (MRE) of 13.8% (Fig 4). Furthermore, model M6 enhanced accuracy at the plot level, exhibiting a mean relative error of 14.9%. In contrast, AGB estimates using pantropical models of Brown [18] and Chave et al. [17] produced substantial errors, with mean relative errors of 34.5% and 85.2%, respectively. Across all plots, a more accurate AGB estimate, with the lowest relative error, was obtained using our plantation model M2, which included DBH and H as predictor variables (Table 8). Except for M2, M8 surpassed all models in AGB estimation accuracy, displaying relative errors (RE) of 16.2% (Table 8). This outcome suggests that the positive and negative mean relative errors (MREs) of M8 were largely counterbalanced among plots, resulting in accurate AGB estimation. Additionally, the magnitude of the errors produced across the plot AGB estimates was notably greater than those produced by the plot estimates for models of Brown [18] and Chave et al. [17].

## Discussion

### Tree biomass and biometric relationships of the sampled trees

The study findings revealed significant correlations between the measured tree aboveground biomass (AGB) and biometric parameters across all the sampled trees. In particular, strong positive relationships were observed between AGB-DBH and AGB-H for both forests (S1a,b,e,f Fig), suggesting that DBH and H could be key indicators of tree biomass accumulation in the study area. These results are consistent with those of previous studies [25,57], which identified stem diameter and height as key factors in determining tree biomass. However, a weak relationship was observed between tree biomass and wood density (ρ) (S1d,h Fig), implying that ρ is not considered a potential predictor variable in the AGB model for the study area. However, numerous studies [15,17,27,51] have reported the significance of wood basic density as a key predictor variable in biomass models. This aligns with findings suggesting that integrating wood density with DBH and H in models can mitigate the influence of environmental factors on AGB estimations in tropical forests. On the other hand, some earlier research has indicated that wood density performs less effectively in biomass modelling [40,58]. This consideration is particularly relevant for reducing the accessibility and expenses associated with wood density data collection.

#### Multispecies allometric models and their performance.

This paper presented site-specific multispecies biomass estimation models based on the destructive approach and data collected from dry Afromontane natural and plantation forests in Ethiopia. Model performance evaluation tests revealed that the selected models provided reliable parameter estimates. Accurate AGB estimates were obtained using site-specific models tailored to each plantation and natural forest. This is in line with research reports indicating that locally developed allometric models are recommended to use and are expected to provide less uncertainty than generic models [17].

Our findings revealed that the model with only DBH as a predictor variable accounted for 88.7% of the AGB variation in plantation and 90.8% in natural forests. When tree height (H) was included in the DBH-alone model, it explained approximately 90% and 95% of the variation in AGB for plantation and natural forests, respectively. This model (M2) generated a lower bias, with a mean prediction error (MPE) of 5.9% for plantation forest and 2.5% for natural forest. This outcome is consistent with prior studies [15,59], which indicate that incorporating height as a predictor can enhance model performance for many tropical tree species. Chave [17] reported that height is a frequently used variable along with DBH, which may reduce errors in AGB estimation. This approach can partially help capture height‒diameter allometry effects for locally developed biomass estimation models [25,60]

Moreover, adding crown area (CA) to the model resulted in significant improvements in model performance (M6 in Table 5) for both forests. In support of our results, reducing uncertainty has been reported in many studies in which crown dimensions were taken into account in biomass models of different tropical forests [42,59]. However, increasing the number of predictors in the model may lead to uncertainty associated with collinearity problems among predictor variables [37,61]. This argument is supported by the results obtained in this study. For example, for plantation datasets, the integration of crown area (CA) in the model significantly enhanced the performance of the model for accurate AGB estimation of individual trees, whereas it produced lower accuracy across-plot AGB than did model M2. Therefore, we opted to select models with fewer parameters and high estimation performance.

We further evaluated the performance of our models over testing data to ensure their accuracy using a split-sample approach [25]. The fact that the RMSE value obtained from the cross-validation test is nearly equivalent to the standard error (SE) of the complete dataset indicates that our best model (M2) is not overfitting. This finding indicates that the ability of M2 to produce accurate AGB estimations is realistic and not artificial. Furthermore, the model’s parameter values in the viable models and the cross-validations remained consistent across subsets of the “test” dataset (Table 7). The regression diagnostic analysis validated the reliability of the parameter estimates in Model M2. This strengthens our case in which the model (M2) is robust for estimating the AGB in the studied ecosystems. Thus, these results indicate that both DBH and tree height serve as key parameters for accurately determining AGB in the study forests. This significant increase in model performance is in line with the general view that stem diameter and tree height are important predictors of AGB, especially for mixed species [25,62]. Therefore, our best multispecies model, M2, can improve the accuracy of biomass assessment protocols for plantation and natural forests at a given site and beyond.

#### Model comparison and importance of site-specific biomass models.

Although generic pantropical models provide several advantages [17], they also introduce higher levels of estimation errors that significantly influence all levels of biomass estimation. These models can lead to both overestimations [17,51] and underestimations [37,63] when applied to the study forests, Wof-Washa plantation and natural forests. Consistent with our findings, significant uncertainty has been reported when the models proposed by Chave [17] are employed on datasets from other dry tropical forests. The variability in AGB estimates arises from differences in species and structural diversity, climatic conditions, disturbances, and the ecological zones present in dry forests [60,64]. In our study, the interplay of environmental factors such as climatic variability, human disturbance, and animal grazing may have affected the growth and productivity of dry Afromontane forests compared with other dry forests [29]. Consequently, trees in specific locations may exhibit unique phylogenetic allometry [64], creating additional uncertainty in biomass estimation when pantropical generic models are used. Our findings highlight the need for developing site-specific models and implementing a rigorous selection process to reduce uncertainty in forest biomass estimation. This is especially important in developing countries like Ethiopia, where few biomass estimation models exist.

### Bias in the plot and landscape biomass estimation

Assessing the bias in biomass estimation linked to allometric models is crucial for reducing bias in AGB estimates. This, in turn, influences forest management, as well as programs, policies, and regulations aimed at mitigating climate change, such as the REDD+ initiative [56]. A few studies [37,59] have evaluated the application of site-specific models to other sites using the same data. However, this approach may introduce bias in model diagnosis and selection processes. To address these concerns, testing fitted allometric models with independent plot-level inventory data is recommended for AGB estimation [56]. However, the uncertainty arising from combining multiple tree species into a single multispecies model with field inventory data has seldom been assessed in dry Afromontane forests in Ethiopia.

In this study, we assessed the performance of both newly developed and some pantropical models for estimating AGB across different plots and landscapes using inventory data from 40 plots of plantation forests at our study site. Our best site-specific multispecies models of plantation forests (M2 under Groups I) provided accurate plot and landscape AGB estimates. This significant increase in model performance is in line with the general view that stem diameter and tree height are important predictors of AGB, especially for mixed species [25,62]. When wood density (ρ) or crown area (CA) were included in the model, bias increased at both the plot level and across-plot AGB estimates. This is consistent with the findings of Abich [40] and Van-Breugel [56], who included ρ in the model and produced a large bias in landscape AGB estimations. Thus, our findings support those of previous studies [40,65], indicating that model evaluation should be based on both statistical inference and the validation of existing theories and knowledge.

Generally, the site- and vegetation-specific models developed in this study could significantly contribute to efforts being made to obtain reliable biomass and C-stock information and support sustainable forest management in dry Afromontane forests of northcentral Ethiopia. Our models have potential applications in other similar ecosystems, particularly in data-limited areas and the scarcity of robust models. Nevertheless, the performance of these models in different ecosystems requires additional verification. Future studies could explore the impact of environmental factors, such as climate and topography, on the effectiveness of various biomass models and examine how different predictor variables influence forest biomass and carbon storage.

## Conclusions

This study has successfully derived robust, local multispecies allometric models for aboveground biomass (AGB) in both plantation and natural dry Afromontane forests of northcentral Ethiopia, demonstrating a significant improvement in accuracy over pan-tropical models. The high explanatory power of these models, accounting for 90% and 95% of AGB variation in plantations and natural forests respectively, underscores their reliability for accurate carbon stock estimation and subsequent forest carbon accounting. The reduced bias achieved by these local models is paramount for precise quantification of ecosystem services, providing a solid foundation for informed forest management decisions. The observed variability in AGB predictions at plot and landscape levels, depending on the model employed, highlights the critical need for context-specific model application. This understanding is essential for refining AGB estimation and ensuring the effectiveness of climate change mitigation strategies, including REDD++ initiatives. The findings of this research contribute directly to enhancing the accuracy of carbon dynamic monitoring, a crucial component in global efforts to combat climate change. Furthermore, the development of integrated multispecies models, incorporating remote sensing and field inventory data across diverse ecosystems in Ethiopia and wider Africa, is imperative. Such advancements will significantly improve regional carbon assessments and provide critical data for sustainable forest management. Ultimately, the advancement of species-and-site-specific models contributes to the broader goal of developing a reliable, generalized global biomass estimation framework, facilitating accurate carbon accounting at regional and global scales. This study serves as a valuable stepping stone towards achieving more precise and comprehensive carbon assessments, vital for effective climate change mitigation and informed environmental stewardship.

## Supporting information

S1 Fig AGB of harvested trees as a function of DBH, height, crown area, and wood density. The black dots in a–d Fig represent harvested sample trees (n = 69) from plantation forest, whilst those from natural forest (n = 58) are represented in e–h Fig.
(TIF)

S1 Table Summary of diameter-class distribution of trees in Wof-Washa Forests. DBH refers to diameter at breast height. N indicates the number of individual trees observed in plantation and natural forests for each diameter class, while the value in % shows the proportion of trees (percentage) within each diameter class. ^*^Trees with a height > 2 m and DBH ≥ 2.5 cm included in the records.
(DOCX)

S1 File Plot inventory data from the Wof-Washa plantation forest in northcentral Ethiopia. DBH, H, and CA refer to diameter at breast height, total tree height, and crown area, respectively. Plot no. represents the number of the squared sample plot, which measures 20m x 20m.
(XLSX)

## References

[1] Wood TE, Cavaleri MA, Reed SC. Tropical forest carbon balance in a warmer world: a critical review spanning microbial- to ecosystem-scale processes. Biol Rev. 2012;87(4):912–27. doi: 10.1111/j.1469-185X.2012.00232.x 22607308

[2] Poorter L, van der Sande MT, Thompson J, Arets EJMM, Alarcón A, Álvarez-Sánchez J, et al. Diversity enhances carbon storage in tropical forests. Glob Ecol Biogeogr. 2015;24(11):1314–28.

[3] Beer C, Reichstein M, Tomelleri E, Ciais P, Jung M, Carvalhais N, et al. Terrestrial gross carbon dioxide uptake: global distribution and covariation with climate. Science. 2010;329(5993):834–8. doi: 10.1126/science.1184984 20603496

[4] Zhou X, Fu Y, Zhou L, Li B, Luo Y. An imperative need for global change research in tropical forests. Tree Physiol. 2013;33(9):903–12. doi: 10.1093/treephys/tpt064 24128847

[5] van der Werf GR, Morton DC, DeFries RS, Olivier JGJ, Kasibhatla PS, Jackson RB, et al. CO2 emissions from forest loss. Nat Geosci. 2009;2(11):737–8. doi: 10.1038/ngeo671

[6] IPCC. IPCC, 2023: Sections. Core Writing Team, Lee H, Romero J, editors. In: Climate Change 2023: Synthesis Report. Contribution of Working Groups I, II and III to the Sixth Assessment Report of the Intergovernmental Panel on Climate Change. IPCC; Geneva. Vol. 13. 2023.

[7] Kim D-G, Chung S-Y, Melka Y, Negash M, Tolera M, Yimer F, et al. Calling for collaboration to cope with climate change in Ethiopia: focus on forestry. J Clim Chang Res. 2018;9(4):303–12.

[8] Birhanu A. Environmental degradation and management in Ethiopian highlands: review of lessons learned. Int J Environ Prot Policy. 2014;2(1):24. doi: 10.11648/j.ijepp.20140201.14

[9] Bekele T. Integrated utilization of eucalyptus globulus grown on the Ethiopian highlands and its contribution to rural livelihood: a case study of Oromia, Amhara and Southern Nations Nationalities and People’s Regional State Ethiopia. International J basic Appl Sci. 2015;4(2):80–7.

[10] Tadesse W, Gezahgne A, Tesema T, Shibabaw B. Plantation forests in Amhara Region: challenges and best measures for future improvements. World J Agric Res. 2019;7(4):149–57.

[11] Gonzalo J, Zewdie S, Tenkir E, Moges Y. REDD+ and carbon markets: The Ethiopian Process. 2017. p. 151–83.

[12] Lemenih M, Allan C, Biot Y. Making forest conservation benefit local communities: participatory forest management in Ethiopia. Vol. 3. NTFP-PFM Research & Development Project The. 2015. p. 12.

[13] Henry M, Picard N, Trotta C, Manlay RJ, Valentini R, Bernoux M, et al. Estimating tree biomass of sub-Saharan African forests: A review of available allometric equations. Silva Fenn. 2011;45(3):477–569.

[14] Ekoungoulou R, Nzala D, Liu X, Niu S. tree biomass estimation in central African forests using allometric models. Open J Ecol. 2018;08(03):209–37.

[15] Basuki TM, Van Laake PE, Skidmore AK, Hussin YA. Allometric equations for estimating the above-ground biomass in tropical lowland Dipterocarp forests. For Ecol Manage. 2009;257(8):1684–94.

[16] Sebrala H, Abich A, Negash M, Asrat Z, Lojka B. Tree allometric equations for estimating biomass and volume of Ethiopian forests and establishing a database: review. Trees Forests People. 2022;9:100314. doi: 10.1016/j.tfp.2022.100314

[17] Chave J, Réjou-Méchain M, Búrquez A, Chidumayo E, Colgan MS, Delitti WBC, et al. Improved allometric models to estimate the aboveground biomass of tropical trees. Glob Chang Biol. 2014;20(10):3177–90. doi: 10.1111/gcb.12629 24817483

[18] Brown. Estimating Biomass and Biomass Change of Tropical Forests: a Primer. (FAO Forestry Paper - 134). FAO For Pap 134. 1997. p. 1–44.

[19] Djomo AN, Picard N, Fayolle A, Henry M, Ngomanda A, Ploton P, et al. Tree allometry for estimation of carbon stocks in African tropical forests. Forestry. 2016;89(4):446–55. doi: 10.1093/forestry/cpw025

[20] Gibbs HK, Brown S, Niles JO, Foley JA. Monitoring and estimating tropical forest carbon stocks: making REDD a reality. Environ Res Lett. 2007;2:13.

[21] Clark DB, Kellner JR. Tropical forest biomass estimation and the fallacy of misplaced concreteness. J Veg Sci. 2012;23(6):1191–6.

[22] Laumonier Y, Edin A, Kanninen M, Munandar AW. Landscape-scale variation in the structure and biomass of the hill dipterocarp forest of Sumatra: Implications for carbon stock assessments. For Ecol Manage. 2010;259(3):505–13.

[23] Pati PK, Kaushik P, Khan ML, Khare PK. Allometric equations for biomass and carbon stock estimation of small diameter woody species from tropical dry deciduous forests: support to REDD+. Trees For People. 2022;9:100289. doi: 10.1016/j.tfp.2022.100289

[24] Sun H, Wang X, Fan D. Effects of climate, biotic factors, and phylogeny on allometric relationships: testing the metabolic scaling theory in plantations and natural forests across China. For Ecosyst. 2020;7(1).

[25] Mokria M, Mekuria W, Gebrekirstos A, Aynekulu E, Belay B, Gashaw T, et al. Mixed-species allometric equations and estimation of aboveground biomass and carbon stocks in restoring degraded landscape in northern Ethiopia. Environ Res Lett. 2018;13(2).

[26] Moges Y, Haile M, Livingstone J. Integration of forest landscape restoration in Ethiopia’s nationally determined contributions 1 Integration of forest landscape restoration in Ethiopia’s nationally determined contributions A review, with a focus on drylands The Pastoral and Environmental. 2021; Available from: www.tropenbos.org

[27] Chave J, Andalo C, Brown S, Cairns MA, Chambers JQ, Eamus D, et al. Tree allometry and improved estimation of carbon stocks and balance in tropical forests. Oecologia. 2005;145(1):87–99. doi: 10.1007/s00442-005-0100-x 15971085

[28] Gebeyehu G, Soromessa T, Bekele T, Teketay D. Carbon stocks and factors affecting their storage in dry Afromontane forests of Awi Zone, northwestern Ethiopia. J Ecol Environ. 2019;43(1):1–18.

[29] Yirga F, Marie M, Kassa S, Haile M. Impact of altitude and anthropogenic disturbance on plant species composition, diversity, and structure at the Wof-Washa highlands of Ethiopia. Heliyon. 2019;5. 10.1016/j.heliyon.2019.e02284 PMC6702390 31453405

[30] Tadesse S, Teketay D. Perceptions and attitudes of local people towards participatory forest management in Tarmaber District of North Shewa Administrative Zone, Ethiopia: the case of Wof-Washa Forests. Ecol Process. 2017;6(1).

[31] Teketay D, Bekele T. Floristic composition of Wof-Washa natural forest, Central Ethiopia: implications for the conservation of biodiversity. Feddes Repert. 1995;106:127–47. doi: 10.1002/fedr.4921060123

[32] Goshme B, Yihune M. Population structure and habitat use of gelada baboon (Theropithecus gelada) in Wof-Washa Forest (Gosh-Meda Area), Central Ethiopia. J Ecol Environ. 2018;42(1):1–6.

[33] Yirga F, Marie M, Kassa S, Haile M. Impact of altitude and anthropogenic disturbance on plant species composition, diversity, and structure at the Wof-Washa highlands of Ethiopia. Heliyon. 2019;5(8):e02284. doi: 10.1016/j.heliyon.2019.e02284 31453405 PMC6702390

[34] Tadesse W, Gezahgne A, Tesema T, Shibabaw B, Tefera B, Kassa H. Plantation forests in Amhara region: challenges and best measures for future improvements. World J Agric Res [Internet]. 2019;7(4):149–57. Available from: http://pubs.sciepub.com/wjar/7/4/5

[35] Tesfaye MA, Gardi O, Anbessa TB, Blaser J. Aboveground biomass, growth and yield for some selected introduced tree species, namely Cupressus lusitanica, Eucalyptus saligna, and Pinus patula in Central Highlands of Ethiopia. 2020;1–18.

[36] Mokria M, Mekuria W, Gebrekirstos A, Aynekulu E, Belay B, Gashaw T, et al. Mixed-species allometric equations and estimation of aboveground biomass and carbon stocks in restoring degraded landscape in northern Ethiopia. For Ecol Manage. 2018;307(2):219–25.

[37] Tetemke BA, Birhane E, Rannestad MM, Eid T. Allometric models for predicting aboveground biomass of trees in the dry afromontane forests of Northern Ethiopia. Forests. 2019;10(12):1–15. doi: 10.3390/f10121114

[38] Ketterings QM, Coe R, Van Noordwijk M, Ambagau’ Y, Palm CA. Reducing uncertainty in the use of allometric biomass equations for predicting above-ground tree biomass in mixed secondary forests. Ecol Manage. 2001;146(1–3):199–209.

[39] Hagazi N, Kebede M, Mokria M, Birhane E, Gebrekirstos A, Bräuning A. Biomass estimation models for Acacia saligna trees in restored landscapes. Environ Res Commun. 2023;5(12).

[40] Abich A, Negash M, Alemu A, Gashaw T. Aboveground biomass models in the Combretum-Terminalia Woodlands of Ethiopia: testing species and site variation effects. Land. 2022;11(6):1–23.36211983

[41] Mokria M, Gebrekirstos A, Aynekulu E, Bräuning A. Tree dieback affects climate change mitigation potential of a dry afromontane forest in northern Ethiopia. Forest Ecol Manage. 2015;344:73–83. doi: 10.1016/j.foreco.2015.02.008

[42] Sileshi GW A critical review of forest biomass estimation models, common mistakes and corrective measures Ecol Manage. 2014;329:237–54.

[43] Teshome M, Torres CMME, Sileshi GW, Mattos PP de, Braz EM, Temesgen H, et al. Mixed-species allometric equations to quantify stem volume and tree biomass in dry Afromontane Forest of Ethiopia. Open J For. 2022;12(03):263–96. doi: 10.4236/ojf.2022.123015

[44] Kelly John F, Beltz BC. A Comparison of Tree Volume Estimation Models for Forest Inventory. Res Pap [Internet]; (SO-233 USDA Forest serve, Southern forest experiment station. New Orleans, Louisiana). 1987. Available from: doi: 10.2737/SO-RP-233

[45] Picard N, Saint-André L, Henry M. Manual for building tree volume and biomass allometric equations: from field measurement to prediction. FAO; Food and Agricultural Organization of the Rome, Italie; 2012.

[46] Kachamba DJ, Eid T, Gobakken T. Above- and belowground biomass models for trees in the miombo woodlands of Malawi. Forests. 2016;7(2).

[47] Mugasha WA, Eid T, Bollandsås OM, Malimbwi RE, Chamshama SAO, Zahabu E, et al. Allometric models for prediction of above- and belowground biomass of trees in the miombo woodlands of Tanzania. Forest Ecol Manage. 2013;310:87–101. doi: 10.1016/j.foreco.2013.08.003

[48] Negash M, Starr M, Kanninen M, Berhe L. Allometric equations for estimating aboveground biomass of Coffea arabica L. grown in the Rift Valley escarpment of Ethiopia. Agrofor Syst. 2013;87(4):953–66.

[49] Koo I, Lee N, Kil RM. Parameterized cross-validation for nonlinear regression models. Neurocomputing. 2008;71(16–18):3089–95. doi: 10.1016/j.neucom.2008.04.043

[50] James G, Witten D, Hastie T, Tibshirani R. An Introduction to Statistical Learning with Applications in R. Springer; New York, NY, USA; 2013. p. 441.

[51] Asrat Z, Eid T, Gobakken T, Negash M. Aboveground tree biomass prediction options for the Dry Afromontane forests in south-central Ethiopia. Ecol Manage [Internet]. 2020;473:118335. Available from: doi: 10.1016/j.foreco.2020.118335

[52] Zeng W, Zhang L, Chen X, Cheng Z, Ma K, Li Z. Construction of compatible and additive individual-tree biomass models for Pinus tabulaeformis in China. Can J For Res. 2017;47:467–75.

[53] Xiang W, Zhou J, Ouyang S, Zhang S, Lei P, Li J, et al. Species-specific and general allometric equations for estimating tree biomass components of subtropical forests in southern China. Eur J For Res. 2016;135(5):963–79.

[54] Mugasha WA, Eid T, Bollandsås OM, Malimbwi RE, Chamshama SAO, Zahabu E, et al. Allometric models for prediction of above- and belowground biomass of trees in the miombo woodlands of Tanzania. Forest Ecol Manage. 2013;310:87–101. doi: 10.1016/j.foreco.2013.08.003

[55] Xiao C, Ceulemans R. Allometric relationships for below- and aboveground biomass of young Scots pines. Ecol Manage. 2004;203(1–3):177–86.

[56] Van-Breugel M, Ransijn J, Craven D, Bongers F, Hall JS. Estimating carbon stock in secondary forests: Decisions and uncertainties associated with allometric biomass models. Ecol Manage [Internet]. 2011;262(8):1648–57. 10.1016/j.foreco.2011.07.018

[57] Rutishauser E, Noor’an F, Laumonier Y, Halperin J, Rufi’ie, Hergoualch K, et al. Generic allometric models including height best estimate forest biomass and carbon stocks in Indonesia. Ecol Manage. 2013;307:219–25. doi: 10.1016/j.foreco.2013.07.013

[58] Aneseyee AB, Soromessa T, Elias E, Feyisa GL. Allometric equations for selected Acacia species (Vachellia and Senegalia genera) of Ethiopia. Carbon Balance Manag. 2021;16(1):1–9. doi: 10.1186/s13021-021-00196-1 34727268 PMC8561847

[59] Mugasha WA, Mwakalukwa EE, Luoga E, Malimbwi RE, Zahabu E, Silayo DS, et al. Allometric Models for Estimating Tree Volume and Aboveground Biomass in Lowland Forests of Tanzania. Int J For Res. 2016;2016.

[60] Hulshof CM, Swenson NG, Weiser MD. Tree height-diameter allometry across the United States. Ecol Evol. 2015;5(6):1193–204. doi: 10.1002/ece3.1328 25859325 PMC4377263

[61] Zianis D, Muukkonen P, Mäkipää R. Biomass and stem volume equations for tree species in Europe. Silva Fenn. 2005:1–63.

[62] Domingues TF, Feldpausch TR, Lloyd J, Lewis SL, Brienen RJW, Gloor E. Tree height integrated into pantropical forest biomass estimates. Biogeosciences. 2014;9:2567–622.

[63] Ubuy M, Eid T, Bollandsås O, Birhane E. Aboveground biomass models for trees and shrubs of exclosures in the drylands of Tigray, northern Ethiopia. J Arid Environ. 2018. doi: 10.1016/j.jaridenv.2018.05.007

[64] Návar J. Allometric equations for tree species and carbon stocks for forests of northwestern Mexico. Ecol Manage. 2009;257(2):427–34.

[65] Picard N, Rutishauser E, Ploton P, Ngomanda A, Henry M. Should tree biomass allometry be restricted to power models? For Ecol Manage [Internet]. 2015;353:156–63. 10.1016/j.foreco.2015.05.035

