# Supplementary figures and images for: Multispecies allometric models for estimating aboveground biomass in plantation and natural dry Afromontane forests in northcentral Ethiopia
Source: PLoS One. 2025 May 7;20(5):e0322025. doi: 10.1371/journal.pone.0322025 (PMC12058031; doi:10.1371/journal.pone.0322025)

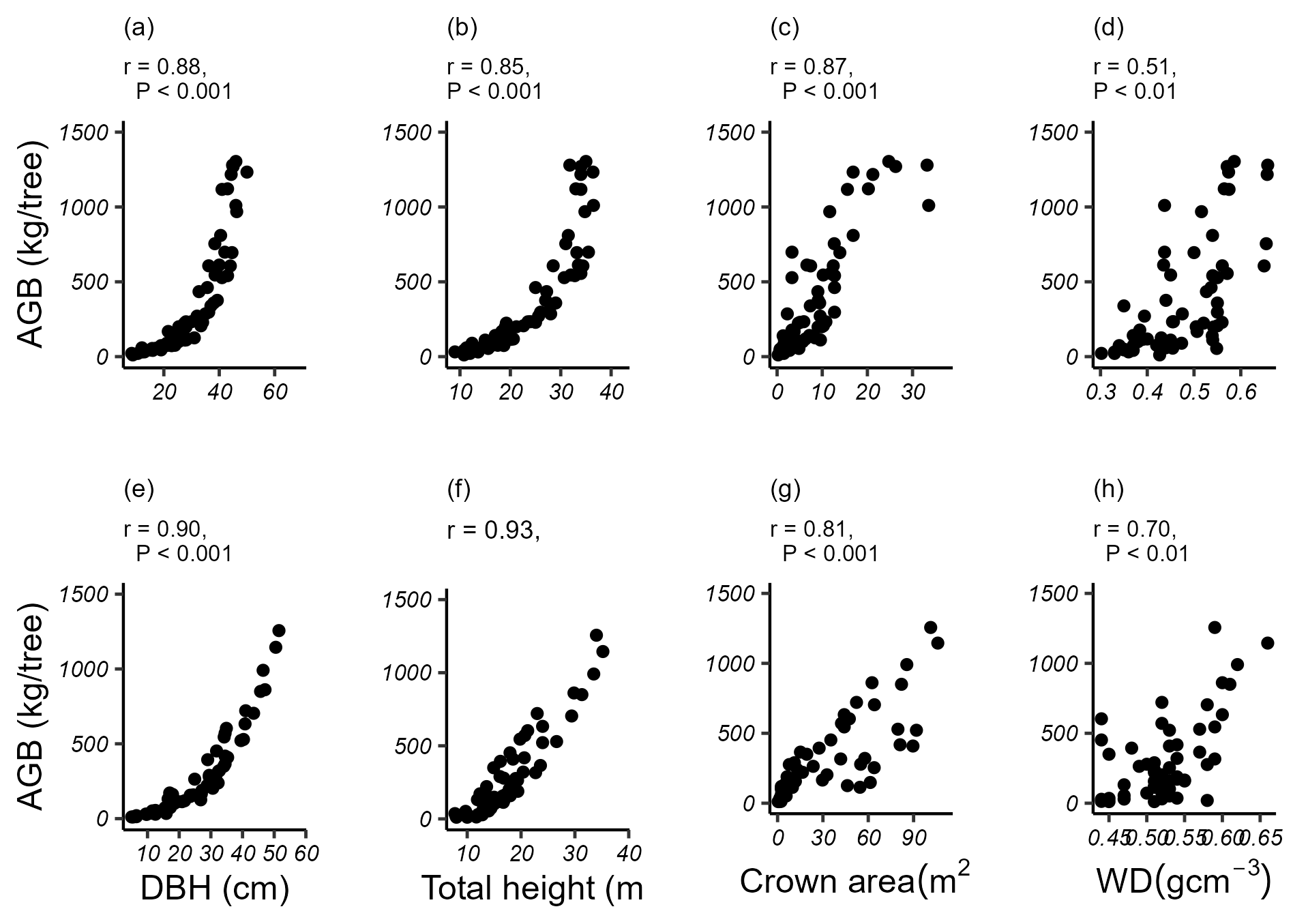

Supplement: S1 Fig — The black dots in a–d Fig represent harvested sample trees (n = 69) from plantation forest, whilst those from natural forest (n = 58) are represented in e–h Fig. (TIF) [file pone.0322025.s001.tif]
