# Supplementary material for: Multispecies allometric models for estimating aboveground biomass in plantation and natural dry Afromontane forests in northcentral Ethiopia
Source: PLoS One. 2025 May 7;20(5):e0322025. doi: 10.1371/journal.pone.0322025 (PMC12058031; doi:10.1371/journal.pone.0322025)
Supplement: S1 Table — DBH refers to diameter at breast height. N indicates the number of individual trees observed in plantation and natural forests for each diameter class, while the value in % shows the proportion of trees (percentage) within each diameter class. *Trees with a height > 2 m and DBH ≥ 2.5 cm included in the records. (DOCX) [file pone.0322025.s002.docx]

| **DBH classes (cm)** | **N (No. of trees)** | | **Total value** | **Value in %** |
| --- | --- | --- | --- | --- |
|  | **N from plantation** | **N from natural forest** |  |  |
| **≤ 10** | 76 | 638 | 714 | 26.24 |
| 10.1–20 | 186 | 468 | 654 | 24.04 |
| **20.1–30** | 284 | 278 | 562 | 20.65 |
| 30.1–40 | 225 | 128 | 353 | 12.97 |
| 40.1–50 | 148 | 75 | 223 | 8.20 |
| ≥ 50.1 | 55 | 160 | 215 | 7.90 |
| Mean | 162.33 | 291.17 | 453.50 | 100 |
| Max | 284.00 | 638.00 | 922.00 |  |
| Min | 55.00 | 75.00 | 130.00 |  |
